# Editorial: The molecular mechanisms of metastasis and therapeutic resistance in breast cancer

**DOI:** 10.3389/fonc.2023.1194858

**Published:** 2023-05-03

**Authors:** Yuanke Liang, Donghong Zhang, Gary M. Tse, Haoyu Lin, Frank A. E. Kruyt

**Affiliations:** ^1^ Department of Thyroid and Breast Surgery, Clinical Research Center, The First Affiliated Hospital of Shantou University Medical College (SUMC), Shantou, China; ^2^ Department of Pharmacology and Chemical Biology, Winship Cancer Institute, Emory University School of Medicine, Atlanta, GA, United States; ^3^ Department of Anatomical and Cellular Pathology, Prince of Wales Hospital, The Chinese University of Hong Kong, Hong Kong, Hong Kong SAR, China; ^4^ Department of Medical Oncology, University of Groningen, University Medical Center Groningen, Groningen, Netherlands

**Keywords:** breast cancer, metastasis (cancer metastasis), therapeutic resistance, molecular mechanisms, EMT - epithelial to mesenchymal transformation

Breast cancer (BC) is the leading cause of cancer-related death among women worldwide (1). The majority of BC-related deaths result from disease recurrence and metastasis, usually related to genetic alterations, epigenetic modification, and cellular plasticity, including cancer stem cells and epithelial-to-mesenchymal transition (EMT) (2–4). This is also a consequence of both intrinsic or acquired resistance to various therapies, such as chemotherapy, endocrine therapy, anti-HER-2 therapy, radiotherapy, and even immunotherapy, which remains a major problem and the most important reason for BC treatment failure (5, 6). Thus, a deeper understanding of the molecular mechanisms of tumor metastasis and therapeutic resistance in BC is needed.

In this Research Topic, the molecular mechanisms of metastasis and therapeutic resistance in BC are presented. We would like to thank all the authors and reviewers who contributed with their relevant works to this Research Topic. In this special issue there are seven articles, as highlighted below.

In the tumor microenvironment, CCL5-CCR5 interaction can lead to BC development and progression. Qiu et al. evaluated the expression of CCL5 in tumor tissue and blood samples in 164 BC patients and found that CCL5 was positively correlated with axillary lymph node metastasis and worse disease-free survival. In addition, clinicopathological correlation analysis showed that CCL5 was highly expressed in HER2-overexpressing and triple-negative breast cancer (TNBC) subtypes. Analysis of peripheral blood and tumor tissue showed that CCL5 promotes BC progression by upregulating the Treg/CD4+CCR5+ cell ratio through CCR5, indicating that CCL5 could be a potential target for BC treatment. Li et al. demonstrated that elevated levels of microvessel density (MVD) in tumors were positively associated with the expression of endothelial-mesenchymal transition (EndMT) markers and worse outcome of BC patients. Using *in vitro* and *in vivo* models, TGF-β was demonstrated to promote proliferation, migration, and angiogenesis mediated by activating p-Smad2/3 and Notch1 signaling. Si et al. have verified that inhibition of RUNX-associated transcription factor 2 (RUNX2) effectively suppressed EMT, proliferation, invasiveness and chemoresistance in MDA-MB-231 epirubicin-resistant cell model. They found that RUNX directly binds to specific motifs in the MMP1 promoter and transactivates its expression, which correlated with BC progression, suggesting that the RUNX2-MMP1 axis could represent biomarkers for diagnosis and treatment.

Chemoresistance remains an important challenge and is responsible for the treatment failure of BC. To explore the mechanism of Doxorubicin (DOX) sensitivity in TNBC, Hum et al. generated chemo-sensitive and chemo-resistant 4T1 syngeneic mouse models. With flow cytometric and single cell transcriptomics analysis, they identified increased IL-17A+ T cells abundance in DOX-sensitive tumors. Furthermore, increased IL-17A levels in TNBC tumor microenvironment correlated with increased responsiveness to DOX and resulted in chronic stimulation of tumor-infiltrating T cells and increased chemosensitivity. In another study by Ansar et al., overexpression of thiosulfate sulfurtransferase–like domain containing 1 (TSTD1) mRNA is correlated with DNA hypomethylation and associated with poor prognosis and resistance to docetaxel, epirubicin and tamoxifen. Moreover, hypomethylation of TSTD1 in cell-free DNA predicted poor chemotherapy efficacy and disease progression in BC patients. This observation indicated that TSTD1 could represent biomarkers for poor prognosis and treatment efficacy in BC. Mou et al. developed patient-derived primary BC cell models *in vitro* to analyze the sensitivity to doxorubicin and pirarubicin. When further validated, this may provide a valuable strategy to predict chemosensitivity *in-vitro* before starting patient chemotherapy in clinic.

Finally, Wang et al. have reviewed the main mechanisms of the primary or acquired resistance to trastuzumab. Several mechanisms were discussed such as ERBB2 gene mutations, transcriptional/post-translational modification of ERBB2, and activation of related signaling pathways in trastuzumab resistance. Importantly, microRNAs, exosomes, bypassing signaling pathways and tumor microenvironment derived signals could serve as biomarkers for predicting trastuzumab resistance in patients. Furthermore, they summarized novel therapeutic agents like tyrosine kinase inhibitors and antibody-drug conjugates that may lay the foundation for additional strategies for targeted therapy in HER2-positive BC.

In summary, this series articles highlights several new insights in the molecular mechanisms involved in BC progression, which helps to deepen our knowledge on metastasis and therapy resistance in BC.

## Author contributions

All authors listed have made a substantial, direct, and intellectual contribution to the work and approved the final manuscript.
